# An intricate vagina penetrating injury with a 22 cm cassava stick in situ for 6 months: a case report

**DOI:** 10.1186/s13256-023-04339-5

**Published:** 2024-01-25

**Authors:** Charles Irumba, Justus Baragaine, Susan Obore, Haruna Mwanje, Julius Nteziyaremye

**Affiliations:** 1 Department of Obstetrics and Gynaecology, College of Health Sciences, Makerere University, Kampala, Uganda; 2 Department of Urogynaecology, Mulago Specialized Women and Neonatal Hospital, Kampala, Uganda; 3 Department of Obstetrics and Gynaecology, Faculty of Health Sciences, Busitema University, Mbale, Uganda; 4 Department of Obstetrics and Gynaecology, Mbale Regional Referral Hospital, Mbale, Uganda

**Keywords:** Vagina penetrating injury, Cassava stick, Case report

## Abstract

**Background:**

Trauma remains one of the major causes of morbidity and mortality and a threat to attainment of sustainable development goal 11. Genital urinary trauma is reported in about 10% of patients presenting with trauma worldwide, and in about 6.6% of patients in Sub-Saharan Africa. If not careful enough, one may miss the foreign body in the vagina and this may be associated with morbidity, and although  rare, mortality.

**Case presentation:**

We report a case of a 7-year-old Black Ugandan that had suffered vagina trauma 6 months prior to presentation at our facility and presented with chronic vagina pus discharge for 6 months. Prior examinations had failed to recognize the foreign body and so did the two abdominal pelvic ultrasound scans. During examination under anesthesia, we were able to locate the cassava stick that had caused penetrating vagina injury and we were able to dislodge it. It was a blunt cassava stick with length of 22 cm and diameter of 2 cm. Although it had gone through the peritoneal cavity, we did not do a laparotomy.

**Conclusion:**

This case emphasizes the need for a thorough vaginal exam including the need to do it under anesthesia with good lighting even when ultrasound scan findings are normal. It presents an opportunity for one to manage penetrating peritoneal injury without a laparotomy in highly selected cases. Gynecologists should be keen as well to rule out child molestation.

## Background

Trauma is a major cause of morbidity globally and the sixth leading cause of death at approximately 10% of all mortalities [1] and therefore a target of sustainable development goal (SDG) 11 of making cities and human settlements inclusive, safe, resilient, and sustainable [2]. There is evidence of an increase in global morbidity and mortality due to trauma. James *et al*. reported increasing trends of trauma cases between 1990 and 2017 from 4,260,493 injury deaths to 4,484,722 deaths in 2017 [3].

Genital urinary tract (GUT) trauma is reported in about 10% of patients presenting with trauma [4]. In a study by Ayun *et al*., after evaluating 21,904 patients presenting with urological emergencies, approximately 6.6% of cases were due to genitourinary trauma [5].

In Uganda, as in many Sub-Saharan African countries, the epidemiology of genitourinary trauma is not well established due to the lack of trauma registries. Most reports are extrapolated from hospital-based data and do not reflect the true incidence. However the rate of GUT is expected to rise in Africa with the increase in motor vehicle accidents, gunshot wounds from civil or domestic conflicts, and recreational activities. This may be compounded by underreporting in Africa since these injuries involve the ‘private’ parts.

Genital trauma may result in external injuries to the labia, vulva or vagina, urethra, and anus and internal injuries to the bony pelvis, bladder, bowels, and reproductive organs [6]. Straddle injuries are the most common cause of genital trauma in pre-pubertal children and occur most often during bicycle riding, falls, and playing on monkey bars [7, 8]. Straddle injuries result, more often than not, as a sequelae of a child straddling an object as they fall, striking the urogenital area with the force of their body weight.

In a young child with genital injury, sexual abuse should not be ignored. Additionally, accidents during recreational activities should be especially considered [6]. In a study by Corey *et al*. regarding the accidental genital trauma (AGT) in girls under the age of 16 years, 70.5% were straddle injuries, followed by non-straddle blunt injuries (23.5%) and penetrating injuries (6.0%). The most common sites of injury were the labia (64.0%), posterior fourchette (7.8%), and hymeneal disruption (8.4%). Encouragingly, 87.9% these were conservatively managed without further complications [9]. If not adequately managed, although they rarely cause mortality, morbidity is quite high.

We present the case of a 7-year-old who had a penetrating vaginal injury by a cassava stick 22 cm in length and 2 cm in diameter that stayed in situ for 6 months. It was associated with chronic vagina pus discharge.

## Case presentation

We present a 7-year-old Black Ugandan primary one pupil from Mpata (landing site on Lake Victoria shores), in Mukono about 35 km away from Mulago Specialized Women and Neonatal Hospital (MSWNH) in Kampala (Uganda’s capital city).

We admitted her at MSWNH on 28 September 2020 through our emergency department following complaints of lower abdominal pain and par vagina pus discharge for 6 months. She came in through Kawempe National referral hospital with a working diagnosis of suspected foreign body in the vagina.

She had been well till about 6 months prior to date of admission. The mother reported that on 4 April 2020 [1 month into the coronavirus disease 2019 (COVID-19) lockdown in Uganda], she sent her daughter to go and fetch water from the lake. While there together with her colleagues, she engaged in recreational activities on the sandy shores. In particular, they fixed cassava sticks of varying height in the sand, over which they would jump into the lake and continue with swimming. They did this repeatedly. As fate may have it, in this particular incident, she failed to jump over one of the sticks, tripped, and saddled onto it. It pierced her vagina and a piece remained in there. She bled profusely from the vagina and then fainted. The older girls with whom she was playing carried her home and narrated the entire story to the mother.

She was taken to the nearby health centre III (largely because they could not go to the hospital due to travel restrictions associated with COVID-19 control). A vaginal pack was applied to control profuse bleeding and she was referred to the general hospital that consequently referred her to one of the national referral facilities.

An abdominal ultrasound scan done, showed normal results, and examination under anesthesia (EUA) followed. However, the family reported that they were informed that nothing had been found.

The child was given ceftriaxone, Ampiclox, paracetamol, and intravenous fluids but no blood transfusion. After 5 days in hospital, the child developed a pus discharge from the vagina. It was foul-smelling and increasing in quantity on a daily basis. There was no passage of stool or leakage of urine from the vagina. The abdominal pain was worsened by ambulation and squatting to urinate, and the paracetamol offered minimal relief. They spent 2 weeks in hospital and were discharged on Ampiclox and paracetamol plus advice of making the child sit in salty water twice daily for 2 weeks.

However, the family reported that despite child initially improving, there were episodes when the pus discharge would worsen and they resorted to keeping her on the previously prescribed medications intermittently depending on the intensity of pus discharge. They report that their efforts to return to the referral hospital were largely hampered by economic shut down and travel restrictions instituted by the government of Uganda.

However, in September 2020 symptoms worsened—the abdominal pain and vaginal pus discharge were unbearable. The child could neither walk nor play. There was loss of appetite and appreciable body weight loss. She also had pain while passing urine but not stool.

They had failed to raise money to return to Kawempe National Referral Hospital because they were no longer selling tomatoes due to the COVID-19 lockdown. When the community members learned of the plight of this child, they collected funds for transport and upkeep and they again traveled to Kawempe National Referral Hospital. When the child was examined, a foreign body in the vagina was suspected and she was referred to MSWNH for the attention of urogynecologists.

Past medical history was unremarkable.

Family and psychosocial history: significant findings were in the psychosocial history as the event was stressful to the family. This was worsened by the COVID-19 travel restrictions.

At MSWNH, we made note of child in fair general condition, afebrile to touch with a temperature of 36.2 °C and wasted (significant weight loss).

*Abdominal examination:* Abdomen was of normal fullness, she had no features of peritonitis, and there were no palpable masses and/or organomegaly. It moved with respiration, had no scars and no therapeutic marks, and was symmetrical. There was no renal angle tenderness. Bowel sounds were heard and normal.

*Vaginal examination:* Vulva was normal but soiled with a foul-smelling pus discharge. Digital vagina examination was deferred due to severe tenderness.

*Rectal examination:* Normal anal skin, no masses, no ulcers, no pus or blood discharge, but was tender to touch. Digital examination was deferred due to severe tenderness.

*Respiratory system:* She had a respiratory rate of 18 breaths per minute. Chest was symmetrical with equal expansion, no scars and trachea was central. Resonant percussion note and normal vesicular breath sounds bilaterally.

*Other systems:* Were grossly normal on examination.

### Diagnostic workup

- Abdominal pelvic ultrasound scan was carried out and it was normal and no foreign bodies were identified.
- Complete blood count was carried out: haemoglobin 10.1 g/dl, mean corpuscular volume (MCV) 87 fL, platelets 560 × 10^3^/µL, and neutrophils count 3.14 × 10^3^/µL
- Upon blood grouping and cross-matching she was found to be O rhesus positive and 1 unit of whole blood was booked for surgery.
- Human immune virus (HIV) antibody test was negative.
- Culture and sensitivity for the pus: we were not able to do this because it was for outsourcing and patient could not afford the service then.
- Diagnostic challenges:
- We were not able to do pus culture and sensitivity.
- Family could not afford an abdominal pelvic computed tomography.
- We were in dilemma but opportunistic since the patient did not have features of peritonitis.
- We made the decision to do examination under anesthesia (EUA) but also had backup option of laparotomy should need arise.

### Findings in surgery

Under aseptic conditions and with the patient under general anesthesia, patient was put in lithotomy position. On examination, we found a normal perineum and external genitalia, with copious pus discharge. On digital vaginal examination, a foreign body was felt lodged in the posterior vaginal wall into the vaginal mucosa. On digital rectal examination, the rectal mucosa felt smooth and mobile with no defect. The foreign body in the vagina extended through a penetrating injury in the posterior fornix and had entered the abdominal cavity through the pouch of Douglas.

**Fig. 1:**
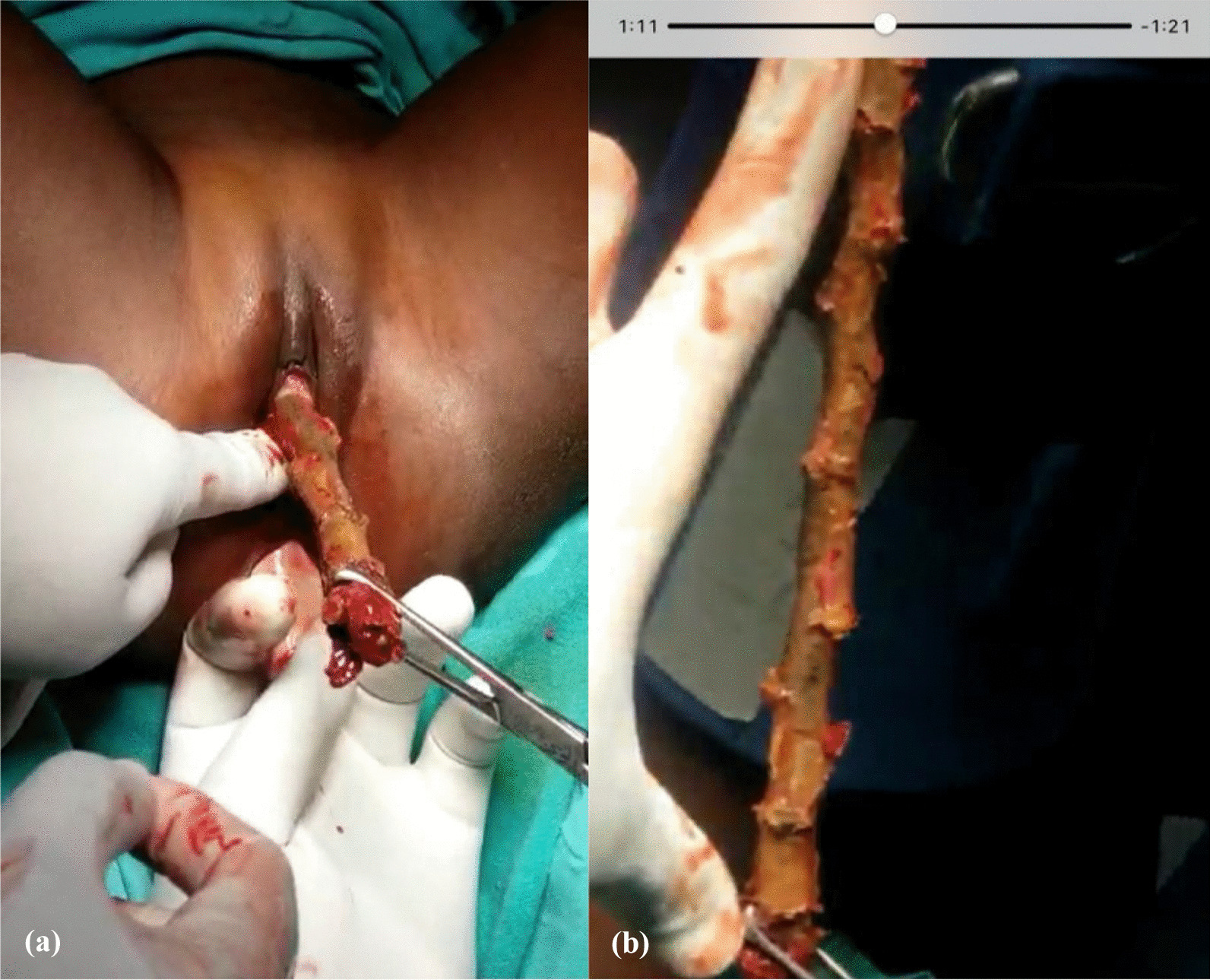
**a** Foreign body (FB) being removed, **b** FB of 22 cm in length and 2 cm in diameter

The foreign body was grasped by Allis tissue forceps and gently pulled out. It was an old cassava stem measuring 22 cm in length and 2 cm in diameter (Fig. 1a, b).

We performed a speculum examination and saw a small hole in the posterior fornix without gut or omentum protruding and there was no active bleeding or pus discharge. The vagina was lavaged with normal saline and iodine and a vaginal pack was inserted. Rectal paracetamol 250 mg was inserted.

Primary diagnosis: penetrating vagina injury.

### Postoperatively

The patient was monitored in the recovery area in surgery for about 1 hour until she was stable, and then transferred to the urogynecology ward. She was awake and breathing well.

We managed her on intravenous antibiotics for 1 week (piperacillin and tazobactam combination 2.25 g daily for 7 days and metronidazole 250 mg every 8 hours for 7 days) and analgesics (rectal paracetamol 250 mg every 8 hours for 5 days)

We did continuous bladder drainage for 24 hours and removed urinary catheter.

We removed vaginal pack after 24 hours.

Patient was admitted for 7 days and there was no evidence of peritonitis and/or pus discharge. Patient remained febrile and freely ambulatory. Review in the clinic 1 week later (2 weeks postop) revealed normal findings.

We discharged her and maintained phone conversations with the family for any concerns, and 3 years down the road, the girl is doing well with no complaints. We reviewed her after 3 weeks postoperative and under light sedation, observed the narrowing to almost complete closure of posterior vagina fornix defect.

## Discussion

We present a case of penetrating vaginal injury in a 7-year-old juvenile patient who had a cassava stick lodged through the posterior vaginal fornix into the abdominal cavity for 6 months with no evident clinical features of gut perforation or peritonitis.

This child had difficulties in accessing timely treatment due to socioeconomic factors compounded by COVID-19 control restrictions.

Trauma is a major cause of morbidity globally and the sixth leading cause of death, accounting for approximately 10% of all mortalities [1]. Furthermore, genital urinary tract (GUT) trauma is reported in about 10% of patients presenting with trauma [4]. Genital trauma may result in external injuries to the labia, vulva or vagina, urethra, and anus and internal injuries to the bony pelvis, bladder, bowels, and reproductive organs [6].

In the African setting, genital injuries are largely reported in conflict and war-torn areas [10], cultural practices such as female genital mutilation/cutting [11], or for ritual sacrifices [12].

The anatomical adaptations of the genitalia during pubertal stage through to the adulthood stage such as well-developed fat pads in the labial area, which are deficient during childhood, predispose to more genital injuries in pre-pubertal females [13]. Genital injuries in this age group may also be due to engaging in more violent and risky games [14]. This is in tandem with our case, which featured a pre-pubertal girl engaged in a case of jumping over cassava sticks that had sharp edges.

Noteworthy, the spatial orientation of cervix to the long axis of vagina predisposes the posterior fornix to injuries [15], as happened in this case.

Although most of the injuries are accidental, minor, and due to blunt trauma such as straddle injuries, some can be of the penetrating type and are caused by sharp objects, pelvic fractures, and sexual abuse, and they may cause serious lacerations and put the life of a patient in jeopardy [14].

Sometimes the physical exam may not find abnormalities. Recently published articles have highlighted the need for subtle history taking as given by the child or adolescent. This at times is the most important factor in determining the etiology of genital injuries stemming from abuse or accidents [16].

Furthermore, penetrating injuries may be sources of foreign bodies in the vagina. Trauma or chronic irritation from a foreign body placed into the vagina causes an incessant foul-smelling vaginal discharge that will persist until the foreign body is removed, as in our case.

This patient had an inanimate foreign body (cassava stick) lodged in her vagina and into the abdominal cavity through a hole in the posterior fornix, causing continuous irritation and a foul-smelling discharge with some associated pruritus and abdominal pain for 6 months.

If a patient has extensive lacerations or severe tenderness, examination under anesthesia should be performed for thorough assessment and to exclude intraperitoneal damage. If the peritoneal cavity has been breached, abdominal cavity exploration by laparotomy or laparoscopy is warranted to exclude visceral injury and supralevator or retroperitoneal hematoma [13, 17].

While genital injuries alone rarely result in death, complications (such as chronic discomfort, dyspareunia, infertility, and or fistula formation) arising from them may cause untoward morbidity and mortality. It is therefore imperative that practitioners, especially gynecologists, take a keen interest in ruling them out, since timely management and follow-up support for the patient’s mental, emotional, and physical well-being need to be addressed [6].

The extent of the injury may be missed by not performing an adequate clinical assessment because of pain or because of a large blood clot partly obscuring the injury. This seemed to be the case in this young girl at the prior two facilities before she came to MSWNH. It is therefore recommended that examination under anesthesia (EUA) with good lighting is carried out [13, 16–18].

It is important to recognize that some injuries to the upper vagina enter the peritoneal cavity and may injure the bowel, bladder, or posterior wall of the uterus. In this case the peritoneal cavity was breached but no evidence of gut perforation was found.

The patient was admitted to the urogynecology ward for examination under anesthesia and foreign body removal. This was successfully done, and it was found that the foreign body had eroded the mucosa of the posterior vaginal wall. It also extended through a penetrating injury in the posterior fornix and had entered the abdominal cavity through the pouch of Douglas.

The foreign body was grasped by Allis tissue forceps and gently pulled out. It was an old cassava stem measuring 22 cm in length and 2 cm in diameter. Speculum examination revealed a small hole in the posterior fornix. There was no protrusion of gut or omentum, no active bleeding, and no pus discharge.

Exploratory laparotomy was deferred since the patient had been fairly stable with the foreign body for 6 months without signs of peritonitis. It was agreed that the patient be monitored on the ward for 5–7 days for any new development of signs of peritonitis. This new development would warrant an exploratory laparotomy to identify injured viscera either from the initial injury or during foreign body removal and retroperitoneal or supralevator hematoma.

The initial treatment goals included achieving hemostasis and restoration of normal anatomy. Irrigation, debridement, and primary repair are key steps during initial management. The vaginal mucosa is typically approximated with running or interrupted stitches with absorbable or delayed-absorbable suture [1]. This was not done for this patient initially just immediately after the injury despite visiting several health facilities.

Presence of infection warrants laceration healing by secondary intention. This patient had a foul-smelling vaginal pus discharge and therefore no suture repair of the vaginal mucosa and the posterior fornix was carried out.

Studies suggest that in cases of penetrating injuries to the genitals, patients should receive a course of broad-spectrum antibiotics and tetanus prophylaxis [19, 20]. This patient received broad-spectrum antibiotics for a full course of 5 days. The choice of piperacillin and tazobactam combination (PISA) and metronidazole was made due to the fact that she had been on and off ceftriaxone and Ampiclox for 6 months. The mother would get refills from drug shops using the initial prescription from Mulago National Referral Hospital. There was no record of this patient receiving tetanus toxoid during the 6 months prior to the removal of the foreign body. This was a missed opportunity in her case management.

## Conclusion

Injuries involving the lower genital tract constitute an emergency that needs keen history taking and thorough examination, including under anesthesia with good lighting. This avoids missing crucial findings that are important in determining patient management. Importantly, a gynecologist should be involved in such case management since they are better trained in the pelvic exam.

## Data Availability

Data concerning this case can be requested form the corresponding author.

## Abbreviation

EUA: Examination under anesthesia
FB: Foreign body
HIV: Human immune virus
MSWNH: Mulago Specialized Women and Neonatal Hospital
PISA: Piperacillin and tazobactam combination
